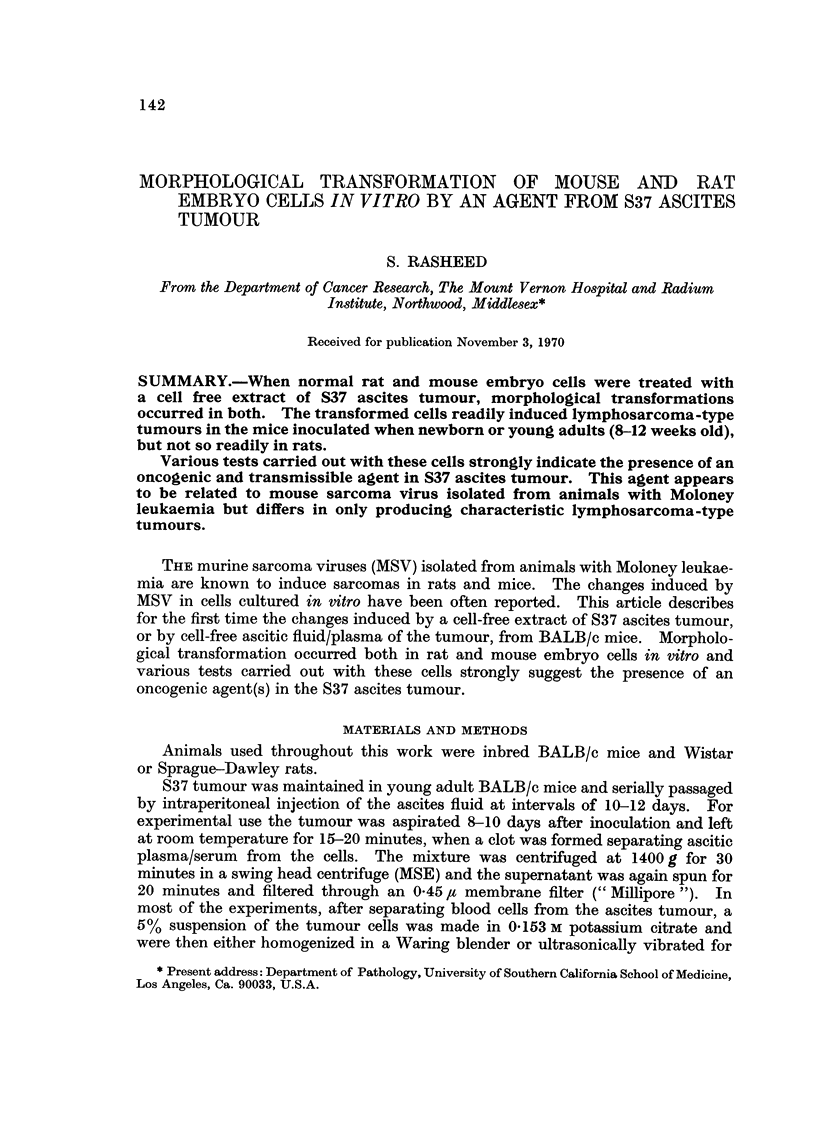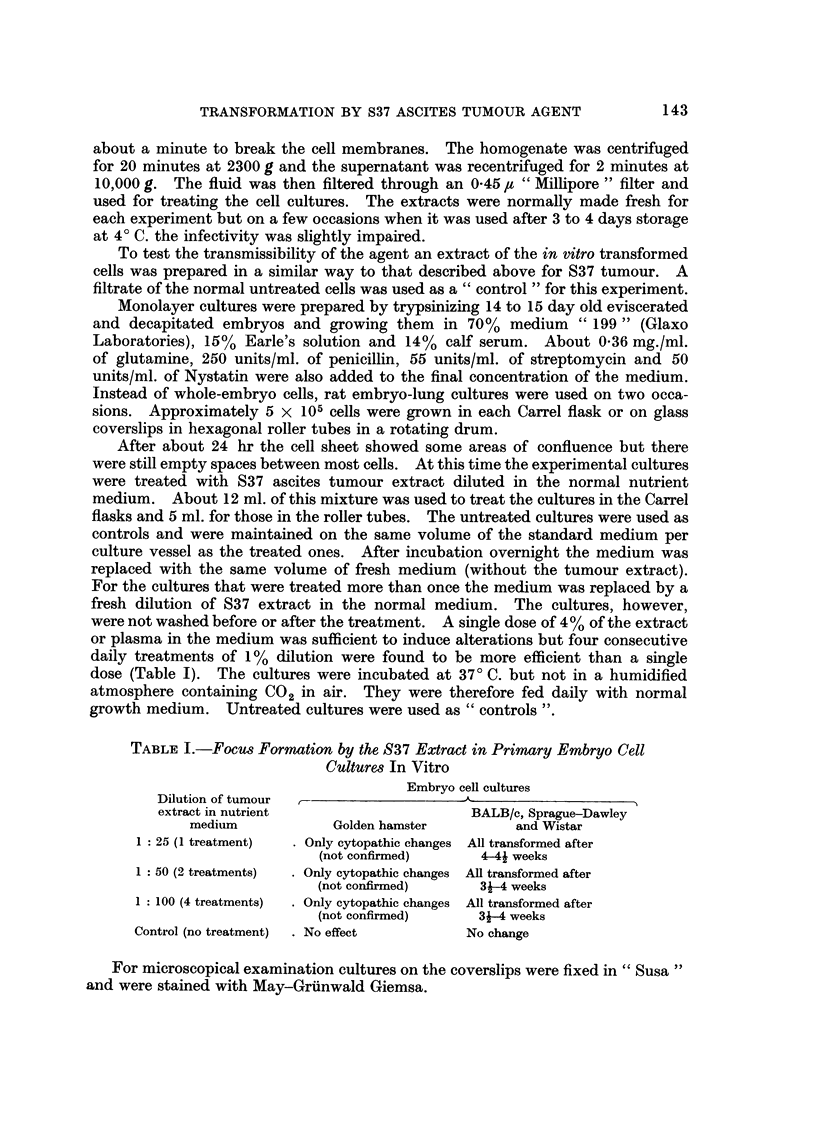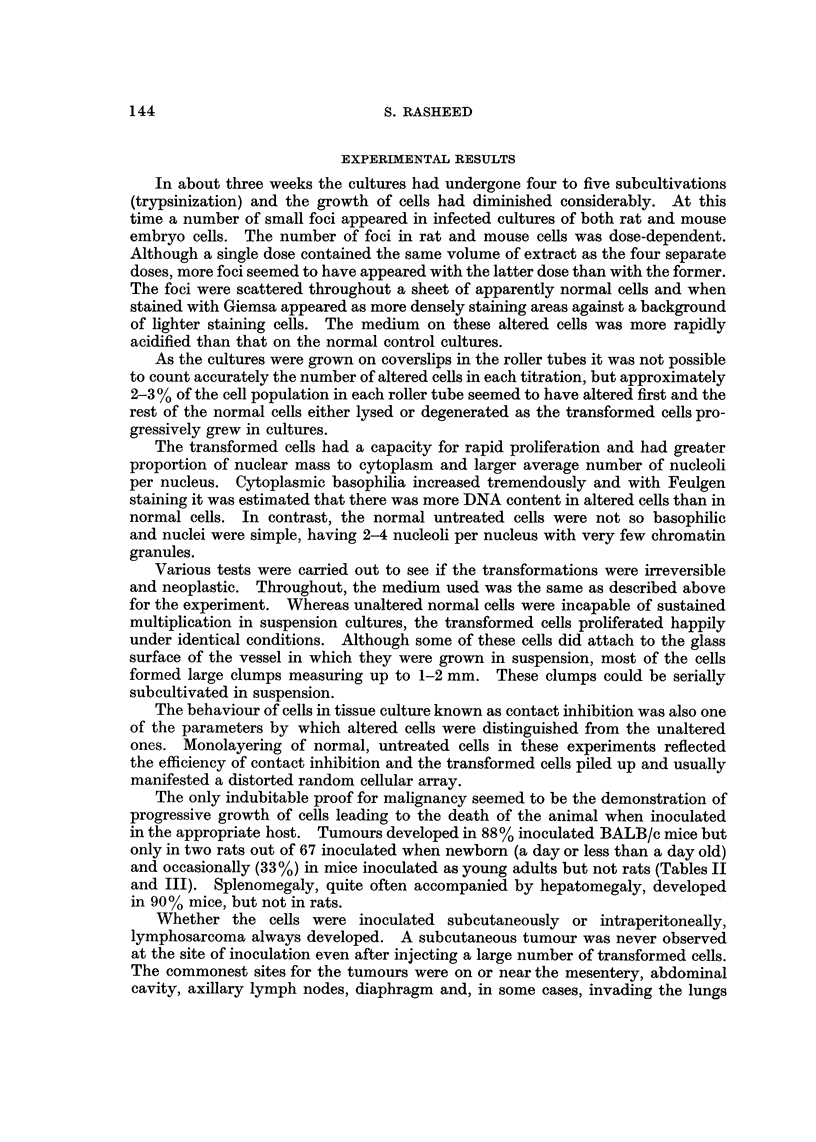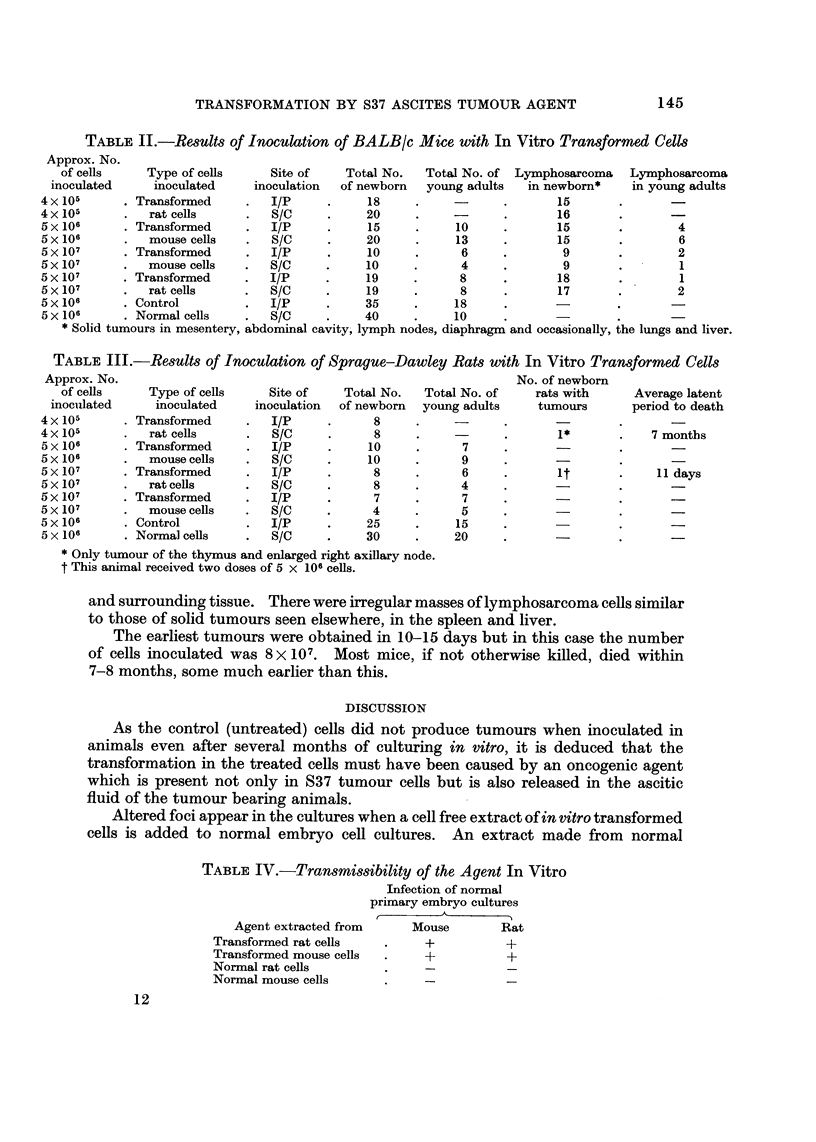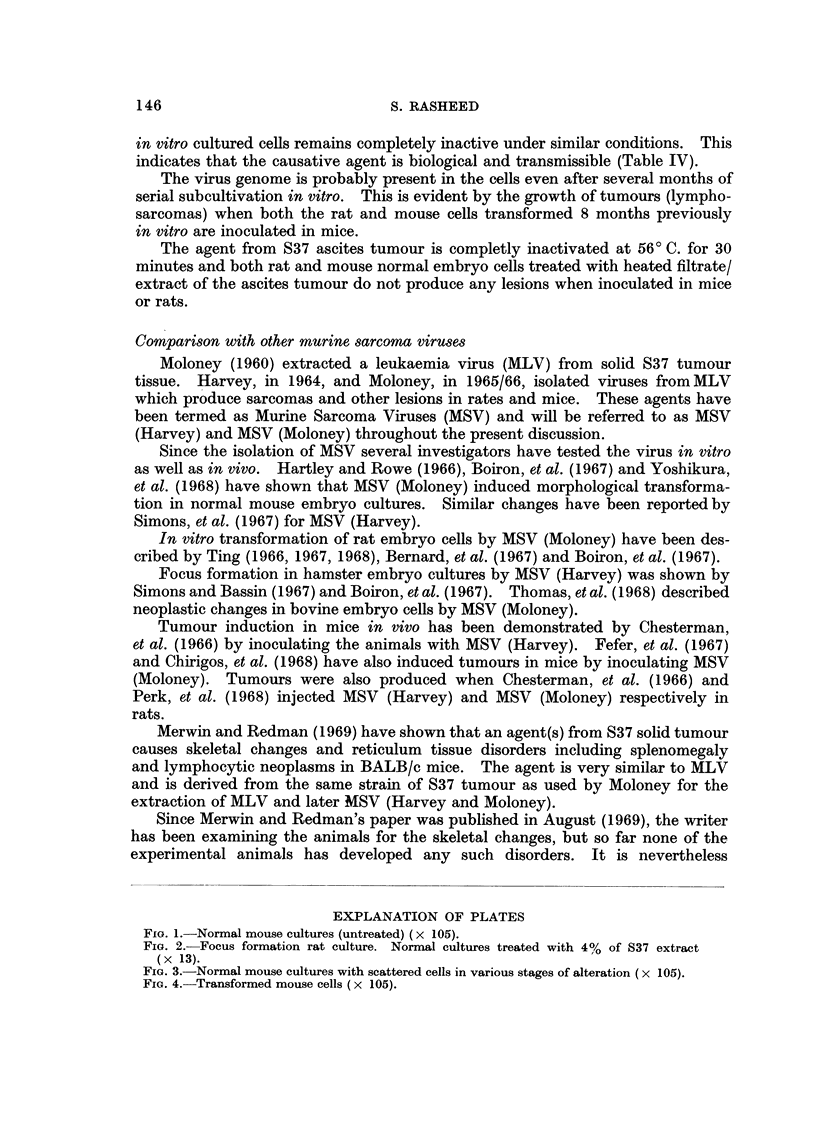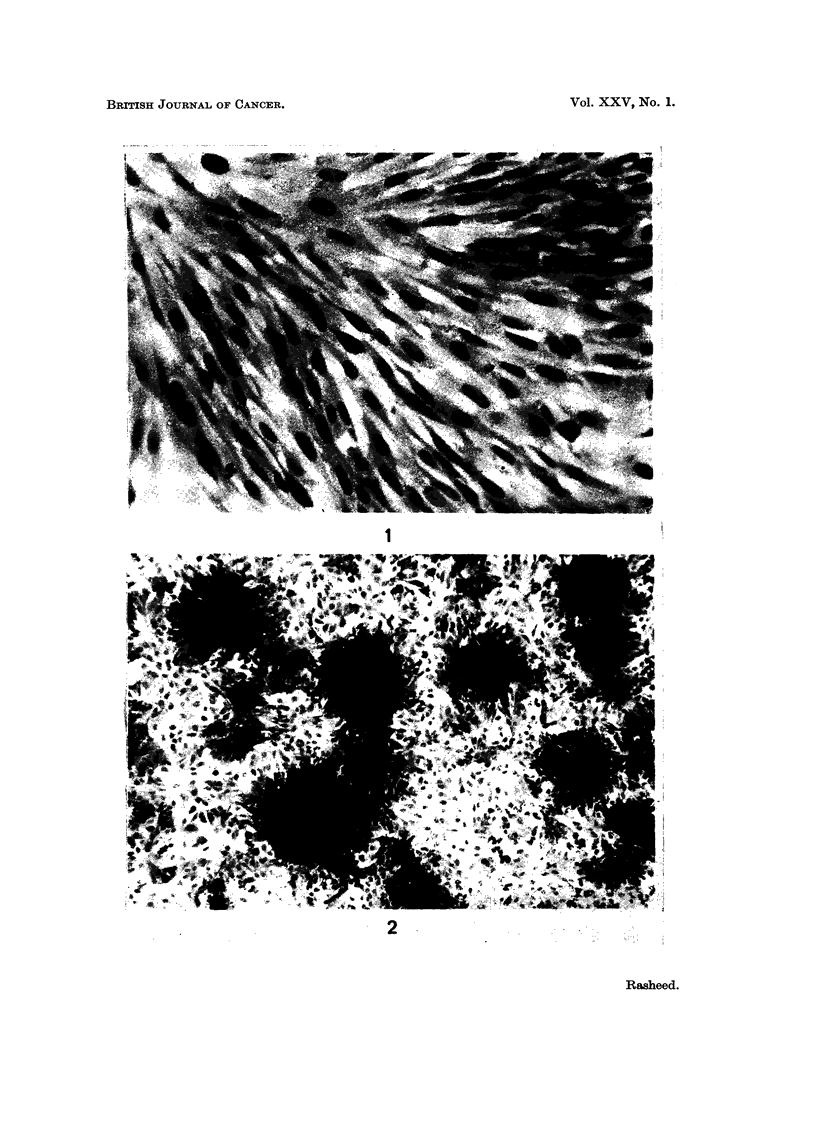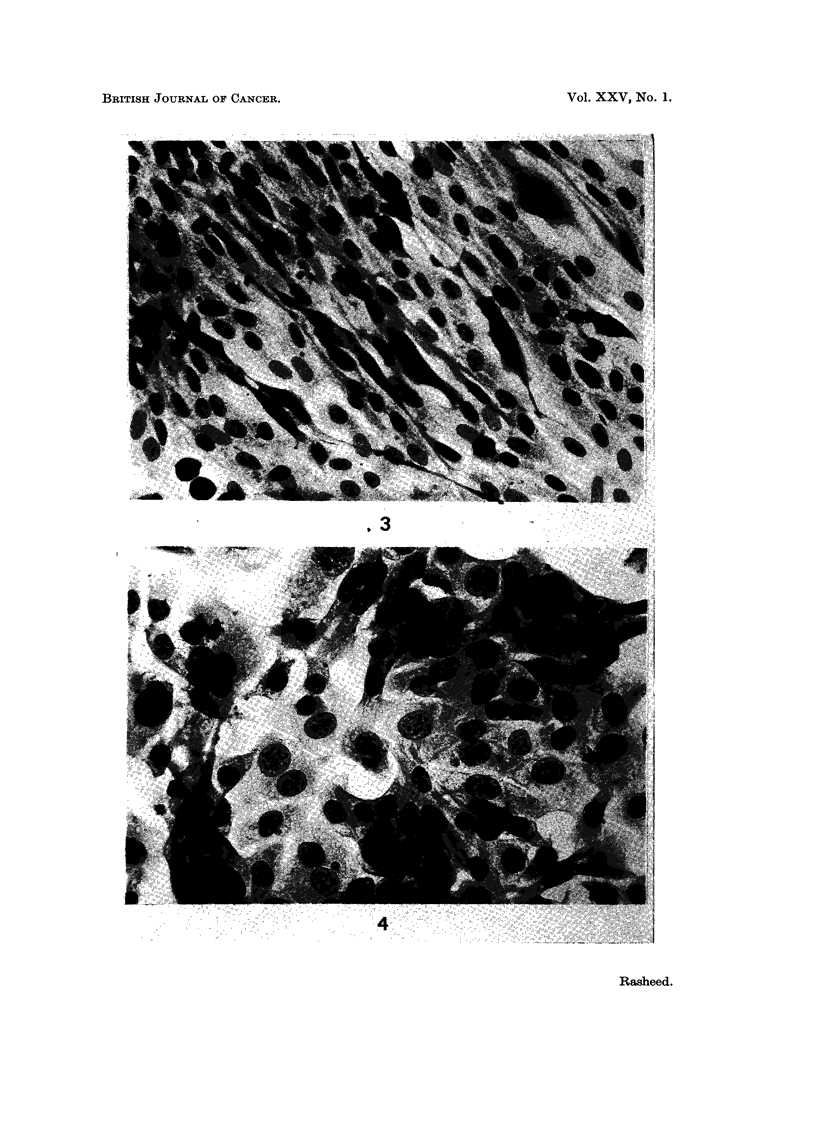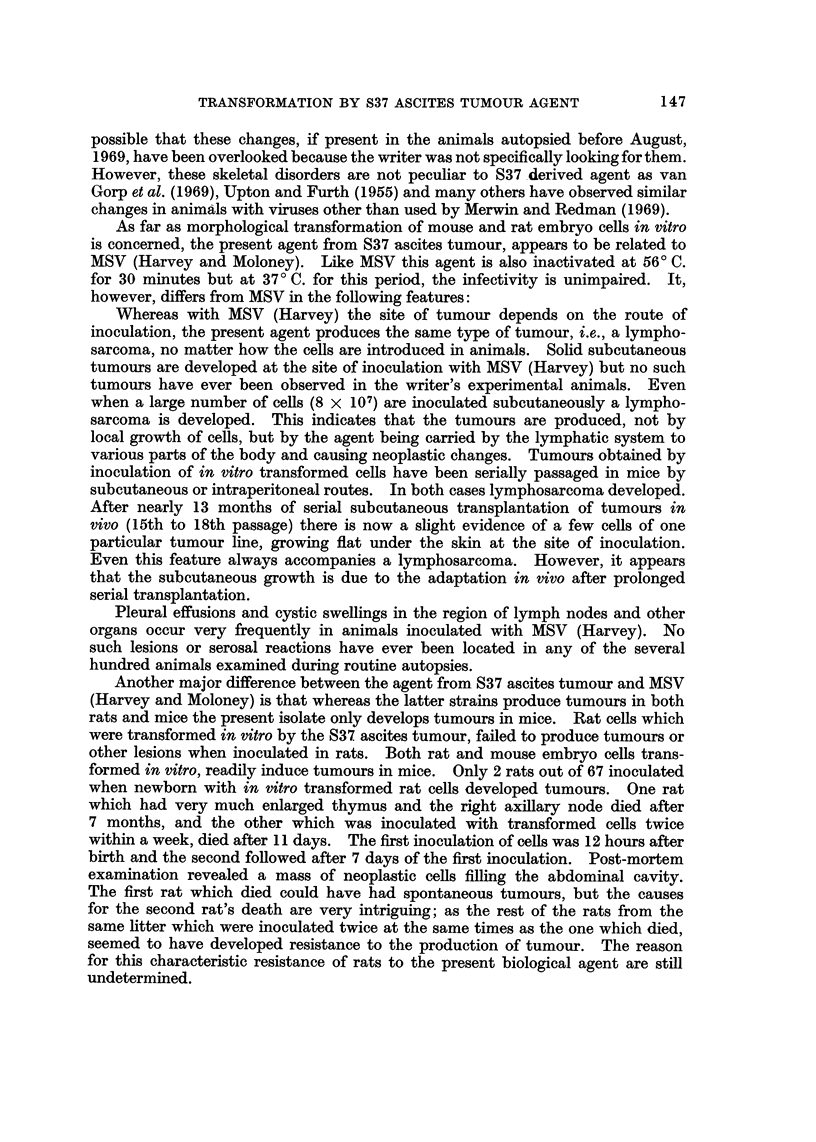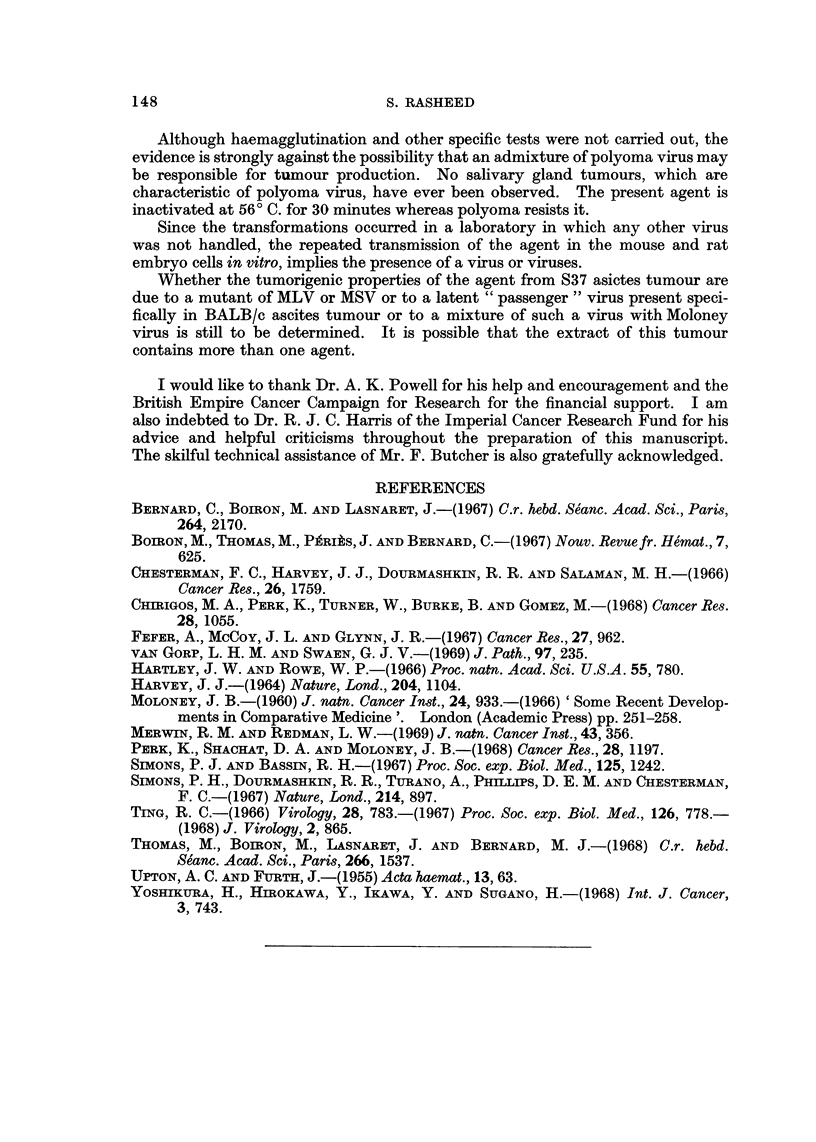# Morphological Transformation of Mouse and Rat Embryo Cells in vitro by an Agent from S37 Ascites Tumour

**DOI:** 10.1038/bjc.1971.20

**Published:** 1971-03

**Authors:** S. Rasheed

## Abstract

**Images:**


### MORPHOLOGICAL TRANSFORMATION OF MOUSE AND RAT EMBRYO CELLS *IN VITRO* BY AN AGENT FROM S37 ASCITES TUMOUR

S. RASHEED

*From the Department of Cancer Research, The Mount Vernon Hospital and Radium Institute, Northwood, Middlesex**

Received for publication November 3, 1970

**SUMMARY.—When normal rat and mouse embryo cells were treated with a cell free extract of S37 ascites tumour, morphological transformations occurred in both. The transformed cells readily induced lymphosarcoma-type tumours in the mice inoculated when newborn or young adults (8–12 weeks old), but not so readily in rats.**

**Various tests carried out with these cells strongly indicate the presence of an oncogenic and transmissible agent in S37 ascites tumour. This agent appears to be related to mouse sarcoma virus isolated from animals with Moloney leukaemia but differs in only producing characteristic lymphosarcoma-type tumours.**

THE murine sarcoma viruses (MSV) isolated from animals with Moloney leukaemia are known to induce sarcomas in rats and mice. The changes induced by MSV in cells cultured *in vitro* have been often reported. This article describes for the first time the changes induced by a cell-free extract of S37 ascites tumour, or by cell-free ascitic fluid/plasma of the tumour, from BALB/c mice. Morphological transformation occurred both in rat and mouse embryo cells *in vitro* and various tests carried out with these cells strongly suggest the presence of an oncogenic agent(s) in the S37 ascites tumour.

### MATERIALS AND METHODS

Animals used throughout this work were inbred BALB/c mice and Wistar or Sprague–Dawley rats.

S37 tumour was maintained in young adult BALB/c mice and serially passaged by intraperitoneal injection of the ascites fluid at intervals of 10–12 days. For experimental use the tumour was aspirated 8–10 days after inoculation and left at room temperature for 15–20 minutes, when a clot was formed separating ascitic plasma/serum from the cells. The mixture was centrifuged at 1400 *g* for 30 minutes in a swing head centrifuge (MSE) and the supernatant was again spun for 20 minutes and filtered through an 0·45 $\mu$ membrane filter (" Millipore "). In most of the experiments, after separating blood cells from the ascites tumour, a 5% suspension of the tumour cells was made in 0·153 M potassium citrate and were then either homogenized in a Waring blender or ultrasonically vibrated for

* Present address: Department of Pathology, University of Southern California School of Medicine, Los Angeles, Ca. 90033, U.S.A.

about a minute to break the cell membranes. The homogenate was centrifuged for 20 minutes at 2300 *g* and the supernatant was recentrifuged for 2 minutes at 10,000 *g*. The fluid was then filtered through an 0·45 $\mu$ " Millipore " filter and used for treating the cell cultures. The extracts were normally made fresh for each experiment but on a few occasions when it was used after 3 to 4 days storage at 4° C. the infectivity was slightly impaired.

To test the transmissibility of the agent an extract of the *in vitro* transformed cells was prepared in a similar way to that described above for S37 tumour. A filtrate of the normal untreated cells was used as a " control " for this experiment.

Monolayer cultures were prepared by trypsinizing 14 to 15 day old eviscerated and decapitated embryos and growing them in 70% medium " 199 " (Glaxo Laboratories), 15% Earle's solution and 14% calf serum. About 0·36 mg./ml. of glutamine, 250 units/ml. of penicillin, 55 units/ml. of streptomycin and 50 units/ml. of Nystatin were also added to the final concentration of the medium. Instead of whole-embryo cells, rat embryo-lung cultures were used on two occasions. Approximately $5 \times 10^5$ cells were grown in each Carrel flask or on glass coverslips in hexagonal roller tubes in a rotating drum.

After about 24 hr the cell sheet showed some areas of confluence but there were still empty spaces between most cells. At this time the experimental cultures were treated with S37 ascites tumour extract diluted in the normal nutrient medium. About 12 ml. of this mixture was used to treat the cultures in the Carrel flasks and 5 ml. for those in the roller tubes. The untreated cultures were used as controls and were maintained on the same volume of the standard medium per culture vessel as the treated ones. After incubation overnight the medium was replaced with the same volume of fresh medium (without the tumour extract). For the cultures that were treated more than once the medium was replaced by a fresh dilution of S37 extract in the normal medium. The cultures, however, were not washed before or after the treatment. A single dose of 4% of the extract or plasma in the medium was sufficient to induce alterations but four consecutive daily treatments of 1% dilution were found to be more efficient than a single dose (Table I). The cultures were incubated at 37° C. but not in a humidified atmosphere containing $CO_2$ in air. They were therefore fed daily with normal growth medium. Untreated cultures were used as " controls ".

TABLE I.—*Focus Formation by the S37 Extract in Primary Embryo Cell Cultures* In Vitro

| Dilution of tumour extract in nutrient medium | Embryo cell cultures: Golden hamster | Embryo cell cultures: BALB/c, Sprague–Dawley and Wistar |
|---|---|---|
| 1 : 25 (1 treatment) | Only cytopathic changes (not confirmed) | All transformed after 4–4½ weeks |
| 1 : 50 (2 treatments) | Only cytopathic changes (not confirmed) | All transformed after 3½–4 weeks |
| 1 : 100 (4 treatments) | Only cytopathic changes (not confirmed) | All transformed after 3½–4 weeks |
| Control (no treatment) | No effect | No change |

For microscopical examination cultures on the coverslips were fixed in " Susa " and were stained with May–Grünwald Giemsa.

### EXPERIMENTAL RESULTS

In about three weeks the cultures had undergone four to five subcultivations (trypsinization) and the growth of cells had diminished considerably. At this time a number of small foci appeared in infected cultures of both rat and mouse embryo cells. The number of foci in rat and mouse cells was dose-dependent. Although a single dose contained the same volume of extract as the four separate doses, more foci seemed to have appeared with the latter dose than with the former. The foci were scattered throughout a sheet of apparently normal cells and when stained with Giemsa appeared as more densely staining areas against a background of lighter staining cells. The medium on these altered cells was more rapidly acidified than that on the normal control cultures.

As the cultures were grown on coverslips in the roller tubes it was not possible to count accurately the number of altered cells in each titration, but approximately 2–3% of the cell population in each roller tube seemed to have altered first and the rest of the normal cells either lysed or degenerated as the transformed cells progressively grew in cultures.

The transformed cells had a capacity for rapid proliferation and had greater proportion of nuclear mass to cytoplasm and larger average number of nucleoli per nucleus. Cytoplasmic basophilia increased tremendously and with Feulgen staining it was estimated that there was more DNA content in altered cells than in normal cells. In contrast, the normal untreated cells were not so basophilic and nuclei were simple, having 2–4 nucleoli per nucleus with very few chromatin granules.

Various tests were carried out to see if the transformations were irreversible and neoplastic. Throughout, the medium used was the same as described above for the experiment. Whereas unaltered normal cells were incapable of sustained multiplication in suspension cultures, the transformed cells proliferated happily under identical conditions. Although some of these cells did attach to the glass surface of the vessel in which they were grown in suspension, most of the cells formed large clumps measuring up to 1–2 mm. These clumps could be serially subcultivated in suspension.

The behaviour of cells in tissue culture known as contact inhibition was also one of the parameters by which altered cells were distinguished from the unaltered ones. Monolayering of normal, untreated cells in these experiments reflected the efficiency of contact inhibition and the transformed cells piled up and usually manifested a distorted random cellular array.

The only indubitable proof for malignancy seemed to be the demonstration of progressive growth of cells leading to the death of the animal when inoculated in the appropriate host. Tumours developed in 88% inoculated BALB/c mice but only in two rats out of 67 inoculated when newborn (a day or less than a day old) and occasionally (33%) in mice inoculated as young adults but not rats (Tables II and III). Splenomegaly, quite often accompanied by hepatomegaly, developed in 90% mice, but not in rats.

Whether the cells were inoculated subcutaneously or intraperitoneally, lymphosarcoma always developed. A subcutaneous tumour was never observed at the site of inoculation even after injecting a large number of transformed cells. The commonest sites for the tumours were on or near the mesentery, abdominal cavity, axillary lymph nodes, diaphragm and, in some cases, invading the lungs

TABLE II.—*Results of Inoculation of BALB/c Mice with* In Vitro *Transformed Cells*

| Approx. No. of cells inoculated | Type of cells inoculated | Site of inoculation | Total No. of newborn | Total No. of young adults | Lymphosarcoma in newborn* | Lymphosarcoma in young adults |
|---|---|---|---|---|---|---|
| $4\times10^5$ | Transformed | I/P | 18 | — | 15 | — |
| $4\times10^5$ | rat cells | S/C | 20 | — | 16 | — |
| $5\times10^6$ | Transformed | I/P | 15 | 10 | 15 | 4 |
| $5\times10^6$ | mouse cells | S/C | 20 | 13 | 15 | 6 |
| $5\times10^7$ | Transformed | I/P | 10 | 6 | 9 | 2 |
| $5\times10^7$ | mouse cells | S/C | 10 | 4 | 9 | 1 |
| $5\times10^7$ | Transformed | I/P | 19 | 8 | 18 | 1 |
| $5\times10^7$ | rat cells | S/C | 19 | 8 | 17 | 2 |
| $5\times10^6$ | Control | I/P | 35 | 18 | — | — |
| $5\times10^6$ | Normal cells | S/C | 40 | 10 | — | — |

* Solid tumours in mesentery, abdominal cavity, lymph nodes, diaphragm and occasionally, the lungs and liver.

TABLE III.—*Results of Inoculation of Sprague–Dawley Rats with* In Vitro *Transformed Cells*

| Approx. No. of cells inoculated | Type of cells inoculated | Site of inoculation | Total No. of newborn | Total No. of young adults | No. of newborn rats with tumours | Average latent period to death |
|---|---|---|---|---|---|---|
| $4\times10^5$ | Transformed | I/P | 8 | — | — | — |
| $4\times10^5$ | rat cells | S/C | 8 | — | 1* | 7 months |
| $5\times10^6$ | Transformed | I/P | 10 | 7 | — | — |
| $5\times10^6$ | mouse cells | S/C | 10 | 9 | — | — |
| $5\times10^7$ | Transformed | I/P | 8 | 6 | 1† | 11 days |
| $5\times10^7$ | rat cells | S/C | 8 | 4 | — | — |
| $5\times10^7$ | Transformed | I/P | 7 | 7 | — | — |
| $5\times10^7$ | mouse cells | S/C | 4 | 5 | — | — |
| $5\times10^6$ | Control | I/P | 25 | 15 | — | — |
| $5\times10^6$ | Normal cells | S/C | 30 | 20 | — | — |

* Only tumour of the thymus and enlarged right axillary node.
† This animal received two doses of $5\times10^6$ cells.

and surrounding tissue. There were irregular masses of lymphosarcoma cells similar to those of solid tumours seen elsewhere, in the spleen and liver.

The earliest tumours were obtained in 10–15 days but in this case the number of cells inoculated was $8\times10^7$. Most mice, if not otherwise killed, died within 7–8 months, some much earlier than this.

### DISCUSSION

As the control (untreated) cells did not produce tumours when inoculated in animals even after several months of culturing *in vitro*, it is deduced that the transformation in the treated cells must have been caused by an oncogenic agent which is present not only in S37 tumour cells but is also released in the ascitic fluid of the tumour bearing animals.

Altered foci appear in the cultures when a cell free extract of *in vitro* transformed cells is added to normal embryo cell cultures. An extract made from normal

TABLE IV.—*Transmissibility of the Agent* In Vitro

| Agent extracted from | Infection of normal primary embryo cultures: Mouse | Rat |
|---|---|---|
| Transformed rat cells | + | + |
| Transformed mouse cells | + | + |
| Normal rat cells | — | — |
| Normal mouse cells | — | — |

*in vitro* cultured cells remains completely inactive under similar conditions. This indicates that the causative agent is biological and transmissible (Table IV).

The virus genome is probably present in the cells even after several months of serial subcultivation *in vitro*. This is evident by the growth of tumours (lymphosarcomas) when both the rat and mouse cells transformed 8 months previously *in vitro* are inoculated in mice.

The agent from S37 ascites tumour is completly inactivated at 56° C. for 30 minutes and both rat and mouse normal embryo cells treated with heated filtrate/extract of the ascites tumour do not produce any lesions when inoculated in mice or rats.

*Comparison with other murine sarcoma viruses*

Moloney (1960) extracted a leukaemia virus (MLV) from solid S37 tumour tissue. Harvey, in 1964, and Moloney, in 1965/66, isolated viruses from MLV which produce sarcomas and other lesions in rates and mice. These agents have been termed as Murine Sarcoma Viruses (MSV) and will be referred to as MSV (Harvey) and MSV (Moloney) throughout the present discussion.

Since the isolation of MSV several investigators have tested the virus *in vitro* as well as *in vivo*. Hartley and Rowe (1966), Boiron, *et al.* (1967) and Yoshikura, *et al.* (1968) have shown that MSV (Moloney) induced morphological transformation in normal mouse embryo cultures. Similar changes have been reported by Simons, *et al.* (1967) for MSV (Harvey).

*In vitro* transformation of rat embryo cells by MSV (Moloney) have been described by Ting (1966, 1967, 1968), Bernard, *et al.* (1967) and Boiron, *et al.* (1967).

Focus formation in hamster embryo cultures by MSV (Harvey) was shown by Simons and Bassin (1967) and Boiron, *et al.* (1967). Thomas, *et al.* (1968) described neoplastic changes in bovine embryo cells by MSV (Moloney).

Tumour induction in mice *in vivo* has been demonstrated by Chesterman, *et al.* (1966) by inoculating the animals with MSV (Harvey). Fefer, *et al.* (1967) and Chirigos, *et al.* (1968) have also induced tumours in mice by inoculating MSV (Moloney). Tumours were also produced when Chesterman, *et al.* (1966) and Perk, *et al.* (1968) injected MSV (Harvey) and MSV (Moloney) respectively in rats.

Merwin and Redman (1969) have shown that an agent(s) from S37 solid tumour causes skeletal changes and reticulum tissue disorders including splenomegaly and lymphocytic neoplasms in BALB/c mice. The agent is very similar to MLV and is derived from the same strain of S37 tumour as used by Moloney for the extraction of MLV and later MSV (Harvey and Moloney).

Since Merwin and Redman's paper was published in August (1969), the writer has been examining the animals for the skeletal changes, but so far none of the experimental animals has developed any such disorders. It is nevertheless

EXPLANATION OF PLATES

FIG. 1.—Normal mouse cultures (untreated) (× 105).
FIG. 2.—Focus formation rat culture. Normal cultures treated with 4% of S37 extract (× 13).
FIG. 3.—Normal mouse cultures with scattered cells in various stages of alteration (× 105).
FIG. 4.—Transformed mouse cells (× 105).

1

2

Rasheed.

3

4

Rasheed.

possible that these changes, if present in the animals autopsied before August, 1969, have been overlooked because the writer was not specifically looking for them. However, these skeletal disorders are not peculiar to S37 derived agent as van Gorp *et al.* (1969), Upton and Furth (1955) and many others have observed similar changes in animals with viruses other than used by Merwin and Redman (1969).

As far as morphological transformation of mouse and rat embryo cells *in vitro* is concerned, the present agent from S37 ascites tumour, appears to be related to MSV (Harvey and Moloney). Like MSV this agent is also inactivated at 56° C. for 30 minutes but at 37° C. for this period, the infectivity is unimpaired. It, however, differs from MSV in the following features:

Whereas with MSV (Harvey) the site of tumour depends on the route of inoculation, the present agent produces the same type of tumour, *i.e.*, a lymphosarcoma, no matter how the cells are introduced in animals. Solid subcutaneous tumours are developed at the site of inoculation with MSV (Harvey) but no such tumours have ever been observed in the writer's experimental animals. Even when a large number of cells ($8 \times 10^7$) are inoculated subcutaneously a lymphosarcoma is developed. This indicates that the tumours are produced, not by local growth of cells, but by the agent being carried by the lymphatic system to various parts of the body and causing neoplastic changes. Tumours obtained by inoculation of *in vitro* transformed cells have been serially passaged in mice by subcutaneous or intraperitoneal routes. In both cases lymphosarcoma developed. After nearly 13 months of serial subcutaneous transplantation of tumours *in vivo* (15th to 18th passage) there is now a slight evidence of a few cells of one particular tumour line, growing flat under the skin at the site of inoculation. Even this feature always accompanies a lymphosarcoma. However, it appears that the subcutaneous growth is due to the adaptation *in vivo* after prolonged serial transplantation.

Pleural effusions and cystic swellings in the region of lymph nodes and other organs occur very frequently in animals inoculated with MSV (Harvey). No such lesions or serosal reactions have ever been located in any of the several hundred animals examined during routine autopsies.

Another major difference between the agent from S37 ascites tumour and MSV (Harvey and Moloney) is that whereas the latter strains produce tumours in both rats and mice the present isolate only develops tumours in mice. Rat cells which were transformed *in vitro* by the S37 ascites tumour, failed to produce tumours or other lesions when inoculated in rats. Both rat and mouse embryo cells transformed *in vitro*, readily induce tumours in mice. Only 2 rats out of 67 inoculated when newborn with *in vitro* transformed rat cells developed tumours. One rat which had very much enlarged thymus and the right axillary node died after 7 months, and the other which was inoculated with transformed cells twice within a week, died after 11 days. The first inoculation of cells was 12 hours after birth and the second followed after 7 days of the first inoculation. Post-mortem examination revealed a mass of neoplastic cells filling the abdominal cavity. The first rat which died could have had spontaneous tumours, but the causes for the second rat's death are very intriguing; as the rest of the rats from the same litter which were inoculated twice at the same times as the one which died, seemed to have developed resistance to the production of tumour. The reason for this characteristic resistance of rats to the present biological agent are still undetermined.

Although haemagglutination and other specific tests were not carried out, the evidence is strongly against the possibility that an admixture of polyoma virus may be responsible for tumour production. No salivary gland tumours, which are characteristic of polyoma virus, have ever been observed. The present agent is inactivated at 56° C. for 30 minutes whereas polyoma resists it.

Since the transformations occurred in a laboratory in which any other virus was not handled, the repeated transmission of the agent in the mouse and rat embryo cells *in vitro*, implies the presence of a virus or viruses.

Whether the tumorigenic properties of the agent from S37 asictes tumour are due to a mutant of MLV or MSV or to a latent " passenger " virus present specifically in BALB/c ascites tumour or to a mixture of such a virus with Moloney virus is still to be determined. It is possible that the extract of this tumour contains more than one agent.

I would like to thank Dr. A. K. Powell for his help and encouragement and the British Empire Cancer Campaign for Research for the financial support. I am also indebted to Dr. R. J. C. Harris of the Imperial Cancer Research Fund for his advice and helpful criticisms throughout the preparation of this manuscript. The skilful technical assistance of Mr. F. Butcher is also gratefully acknowledged.

### REFERENCES

Bernard, C., Boiron, M. and Lasnaret, J.—(1967) *C.r. hebd. Séanc. Acad. Sci., Paris*, **264**, 2170.

Boiron, M., Thomas, M., Périès, J. and Bernard, C.—(1967) *Nouv. Revue fr. Hémat.*, **7**, 625.

Chesterman, F. C., Harvey, J. J., Dourmashkin, R. R. and Salaman, M. H.—(1966) *Cancer Res.*, **26**, 1759.

Chirigos, M. A., Perk, K., Turner, W., Burke, B. and Gomez, M.—(1968) *Cancer Res.* **28**, 1055.

Fefer, A., McCoy, J. L. and Glynn, J. R.—(1967) *Cancer Res.*, **27**, 962.

van Gorp, L. H. M. and Swaen, G. J. V.—(1969) *J. Path.*, **97**, 235.

Hartley, J. W. and Rowe, W. P.—(1966) *Proc. natn. Acad. Sci. U.S.A.* **55**, 780.

Harvey, J. J.—(1964) *Nature, Lond.*, **204**, 1104.

Moloney, J. B.—(1960) *J. natn. Cancer Inst.*, **24**, 933.—(1966) ' Some Recent Developments in Comparative Medicine '. London (Academic Press) pp. 251–258.

Merwin, R. M. and Redman, L. W.—(1969) *J. natn. Cancer Inst.*, **43**, 356.

Perk, K., Shachat, D. A. and Moloney, J. B.—(1968) *Cancer Res.*, **28**, 1197.

Simons, P. J. and Bassin, R. H.—(1967) *Proc. Soc. exp. Biol. Med.*, **125**, 1242.

Simons, P. H., Dourmashkin, R. R., Turano, A., Phillips, D. E. M. and Chesterman, F. C.—(1967) *Nature, Lond.*, **214**, 897.

Ting, R. C.—(1966) *Virology*, **28**, 783.—(1967) *Proc. Soc. exp. Biol. Med.*, **126**, 778.—(1968) *J. Virology*, **2**, 865.

Thomas, M., Boiron, M., Lasnaret, J. and Bernard, M. J.—(1968) *C.r. hebd. Séanc. Acad. Sci., Paris*, **266**, 1537.

Upton, A. C. and Furth, J.—(1955) *Acta haemat.*, **13**, 63.

Yoshikura, H., Hirokawa, Y., Ikawa, Y. and Sugano, H.—(1968) *Int. J. Cancer*, **3**, 743.